# Influenza Vaccination among Patients with Diabetes or Ischemic Heart Disease in Thailand: Coverage, Knowledge and Associated Factors †

**DOI:** 10.3390/vaccines11040794

**Published:** 2023-04-04

**Authors:** Surarong Chinwong, Suthinee Taesotikul, Duangruthai Koenkaew, Thanannat Thanomjit, Arintaya Phrommintikul, Dujrudee Chinwong

**Affiliations:** 1 Department of Pharmaceutical Care, Faculty of Pharmacy, Chiang Mai University, Chiang Mai 50200, Thailand; surarong@gmail.com (S.C.);; 2 Center of Excellence for Innovation in Analytical Science and Technology for Biodiversity-Based Economic and Society (I-ANALY-S-T_B.BES-CMU), Chiang Mai University, Chiang Mai 50200, Thailand; 3 Pharmaceutical Care Training Center (PCTC), Faculty of Pharmacy, Chiang Mai University, Chiang Mai 50200, Thailand; 4 Department of Internal Medicine, Faculty of Medicine, Chiang Mai University, Chiang Mai 50200, Thailand

**Keywords:** influenza vaccination, Thailand, knowledge, coverage, elderly, noncommunicable disease, diabetes, hypertension

## Abstract

An influenza vaccination is recommended for patients with diabetes mellitus (DM) or ischemic heart disease (IHD) to prevent cardiovascular events, but the vaccination coverage remains low. This cross-sectional study sought to investigate vaccination coverage, knowledge level on influenza and factors associated with influenza vaccination in patients with DM or IHD treated at a tertiary hospital in northern Thailand. Patients were interviewed from August to October 2017. Of 150 patients interviewed (51.3% women, mean age of 66.7 ± 8.3 years, 35.3% DM, 35.3% IHD, 29.3% DM and IHD), 45.3% (68/150) were vaccinated against influenza. The mean knowledge score was 9.68 ± 1.35 (total: 11) and did not differ between those receiving the immunization and those who did not (*p* = 0.056). Two factors remained significantly associated with their vaccination after multivariable logistic regression analysis: knowing their right to receive free vaccinations (adjusted OR 2.32, 95% CI: 1.06–5.10, *p*-value: 0.035), and needing to be vaccinated (adjusted OR 3.50, 95% CI: 1.51–8.12, *p*-value: 0.003). Overall, the vaccine coverage was low; less than one-half of patients received the influenza vaccine, but their knowledge level was high. Possessing the right and having a need were two factors associated with vaccination. Such factors should be carefully considered to encourage patients with DM and IDH to receive the influenza vaccination.

## 1. Introduction

Influenza viruses are crucial pathogens for respiratory tract infection. The severity of illness ranges from asymptomatic to severe disease. The aims of the influenza vaccine are to reduce seasonal influenza outbreaks, attenuate the severity of illness and prevent consequences from viral infections. These include acute respiratory distress syndrome, worsening chronic diseases, especially cardiovascular events by increased inflammation process, enhanced prothrombotic activity and increased metabolic demand [1,2].

The World Health Organization (WHO) recommends that patients at a high risk of severe influenza viral infection, such as the elderly (65 years or older) and those with comorbidities such as chronic cardiovascular disease, diabetes and pulmonary disease, receive influenza vaccination [3]. This recommendation is supported by the benefits of influenza vaccination among patients with high cardiovascular risk and cardiovascular diseases by significantly reducing all-cause death, cardiovascular death and major adverse cardiovascular events, including myocardial infarction, coronary revascularization, stroke and hospitalization due to heart failure [4,5]. Influenza vaccines also provide benefits on preventing hospitalization and reducing mortality among patients with diabetes in systematic reviews and meta-analyses [6,7].

In Thailand, the influenza vaccine has been proven cost effective for preventing cardiovascular events and viral pneumonia [8]. Since 2008, eligible patients have been allowed to obtain free influenza vaccines from public or private physicians participating in the National Health Insurance Program [9]. Despite the existence of this program for over a decade, influenza immunization rates among Thai citizens remain unsatisfactory. Influenza remains an economic burden among older adults with chronic illnesses. The high cost of treatment stems from nonvaccinated status, severe influenza pneumonia with complications and hospitalization in an inpatient setting [10]. A recent study in Thailand reported that vaccine coverage among patients with DM remained inadequate with only 50% receiving the influenza vaccination [11].

Although the trend to influenza vaccine coverage has improved in recent years due to health policies and the effort from health care providers [9,10], many patients with chronic diseases, especially those with DM or IHD, have not received an influenza vaccination. Thus, this cross-sectional study aimed to determine influenza vaccination coverage, knowledge level on influenza and factors associated with influenza vaccination among patients with DM or IHD.

## 2. Materials and Methods

### 2.1. Study Population and Samples

This was a cross-sectional study. Participants were patients aged at least 18 years old with DM and/or IHD treated at a tertiary hospital in northern Thailand.

The sample size was determined based on prior study by Owusu JT et al. [12], which reported that 12 to 30 percent of patients with chronic disease were vaccinated against seasonal influenza. We calculated the sample size for estimating a single proportion based on this study with 5% absolute precision and 95% confidence; the sample size of 163 to 355 patients was required.

This study was approved by the Human Research Ethics Committee, Faculty of Medicine, Chiang Mai University, Thailand with approval number 232/2017 and registration number HOS-2560-04503.

### 2.2. Interview-Question Development and Data Collection

The questions and choices were designed using a literature review to answer all the objectives covering vaccination coverage, knowledge levels on influenza and factors associated with the influenza vaccination among patients with DM or IHD. The content validity of the interviewing questions was reviewed by three specialists in the field. The index of Item-Objective Congruence (IOC) was constructed using values ranging from −1 to +1 (+1, congruent; 0 doubtful; and −1, incongruent) to determine whether each question and answer was correct and relevant to the aims of the study. Questions with an IOC of at least 0.5 were retained, while those with an IOC of less than 0.5 were eliminated or modified following consultation. Finally, the questions were tested in 20 patients to approve the language and understandability.

The interview questions included four sections: general information, vaccination coverage, knowledge level of influenza and factors associated with the influenza vaccination (as shown in the Supplement 1). First, general information included sex, age, education level and comorbidities (DM, IHD). Second, a question was asked whether a patient had been vaccinated for influenza. Third, 11 items were asked about their knowledge level of influenza; the two responses were yes or no. Fourth, seven factors associated with influenza vaccination were investigated, with two item choices of yes or no.

Data were collected face-to-face by interviewing patients from August to October 2017; two trained researchers interviewed the patients while they were waiting for their dispensed medication. Each patient was asked to voluntarily provide his/her consent before being interviewed.

### 2.3. Statistical Analysis

Participants’ data were presented using descriptive statistics, means ± standard derivations (SD) for continuous variables and frequencies and percentages for categorical data. Coverage of influenza vaccination was presented in frequencies and percentages and 95% confidence intervals. The correctness of knowledge level about influenza was presented in frequencies and percentages of each item and the mean score of the two groups (vaccinated and unvaccinated). The two groups were compared using a Fisher’s exact test for categorical data and independent t-test for continuous variables. Factors associated with influenza vaccination were analyzed using univariable and multivariable logistic regression and presented in crude and adjusted Odds Ratio (OR) with 95% confidence intervals (95% CI). Variables analyzed in the univariable analysis included personal characteristics of patients, knowledge score (total of 11) on influenza, divided in two groups, high (10/11) and low (<10), and other perception factors on influenza vaccination. Factors with a *p*-value < 0.1 from the univariable analysis were included in the multivariable logistic regression to determine associated factors with influenza vaccinations. Factors with *p*-value < 0.05 were considered statistically significant. STATA Software, Version 14.0, was used to analyze data.

## 3. Results

Of 150 patients with DM or IHD treated at a tertiary hospital in northern Thailand (51.3% women, mean age of 66.7 ± 8.3 years, 35.3% DM, 35.3% IHD, 29.3% DM and IHD), the coverage of influenza vaccination was 45.3% (68/150), 95% confidence interval: 37.3 to 53.3%. We found no differences in coverage of the influenza vaccination (*p*-value > 0.05) between males and females, patients aged <65 and ≥65 years and patients with DM, IHD and DM and IHD (Table 1).

Knowledge of influenza was quite high: the mean knowledge score was 9.68 ± 1.35 (total: 11), and this was not different between patients who had received the influenza vaccination and those who had not, 9.91 ± 1.27, and 9.49 ± 1.40, (*p* = 0.056), respectively. Patients with higher knowledge scores were more likely to be vaccinated than those with lower scores. Patients’ perceptions and experiences regarding influenza vaccination differed between the two groups regarding three factors: knowing the right to receive free vaccination, needing to receive a flu vaccination and experiences in receiving influenza vaccination (Table 1).

Univariable logistic regression revealed six factors associated with influenza vaccination (*p* < 0.1): age higher than 65, a higher score of influenza knowledge, knowing the immunization advantages, knowing the right to be freely vaccinated, needing to be vaccinated and experiences of influenza vaccination. However, only two factors remained significantly associated with their vaccination after the multivariable analysis: knowing their right to receive free vaccinations (adjusted OR 2.32, 95% CI: 1.06 to 5.10, *p*-value: 0.035) and needing to be vaccinated (adjusted OR 3.50, 95% CI: 1.51 to 8.12, *p*-value: 0.003) (Table 2).

## 4. Discussion

### 4.1. Influenza Vaccination Coverage

Influenza vaccine uptake was 45.3% among patients with DM or IHD in our study. The vaccine coverage tended to be higher among patients older than 65 (62%) than among younger subjects (38%). However, the vaccine coverage was still lower than the 75% targeted by the World Health Assembly (resolution WHA 56.19) to reduce the influenza pandemics [13]. Countries that have achieved the WHO target of vaccine coverage among the elderly include England, the USA, and South Korea, with Italy, France, the Netherlands and Australia having incremental increases in vaccine coverage in recent years [14]. In Asia, vaccine coverage among high-risk populations such as the elderly and patients with chronic illness differed by a median of 37.3%, ranging from 0 to 81.7% (from 2008 to 2018) [15]. In Thailand, the electronic database from The Thailand National Health Security Office reported that overall influenza vaccine coverage was low (ranging from 12% to 30%) among individuals with chronic diseases since 2012 [12]. Our result revealed improved influenza vaccine uptake among patients with DM or IHD in northern urban areas revealing a similar vaccine coverage with one related study in Bangkok (52.2%) [11].

### 4.2. Knowledge Score of Influenza

The knowledge score of influenza was high, and the mean scores of knowledge did not differ between those patients who had received the vaccine and those who had not. Vaccinated participants showed higher knowledge scores in most items than unvaccinated patients, although without significant difference between the two groups (as shown in Table S1 indicate that patients in vaccinated and unvaccinated groups have good knowledge scores of the influenza virus and an understanding of clinical presentations, self-management and prevention.

### 4.3. Factors Associated with Influenza Vaccination

After multivariable logistic regression analysis, two factors remained associated with influenza vaccination: knowing their right to receive free vaccinations and perceptions about needing to be vaccinated. These factors must be carefully addressed to urge patients to have influenza vaccinations.

Despite the fact that Thailand’s health program has provided free influenza immunization for the elderly and those with chronic illnesses since 2008 [9], this information and the right to access the vaccine are not well known through the population. An observational study among Thais, conducted by Phrommintikul A, also supported our results that the most common reason of missing annual influenza immunization was lack of motivation to be vaccinated [16]. Therefore, healthcare providers and health systems should promote annual influenza vaccination programs through annual reminders and national media campaigns.

Although the patients in our study showed a high level of knowledge of influenza vaccination, having higher knowledge levels of influenza only showed a tendency to be associated with being vaccinated (*p* = 0.066). Our studies align with other studies that also found patients to be knowledgeable about influenza and vaccination had higher vaccination coverage [17,18,19].

In recent years, due to the COVID-19 outbreak, infodemic and misinformation of disease as well as efficacy and safety of vaccines could be obtained from the Internet or social media. The information overload and information bias from receiving negative feedback regarding vaccines might lead to lowered vaccine coverage [20]. The results from a study of health literacy from older Thais revealed that health information accessed from health care providers, health care volunteers and health network partners still correlated with good health literacy in rural Thais in northern areas [21]. One study conducted by Phrommintikul A also confirmed that good perceptions of influenza vaccination could be motivated by their physicians [16]. The differences between influenza vaccination and COVID-19 vaccination were as follows: (1) The COVID-19 vaccine presented more dynamic situations of safety and efficacy than the influenza vaccine, which has more evidence of the benefits and cost effectiveness. (2) Confidence in the health care system and the quality of new vaccine platforms might affect someone’s decision to receive the COVID-19 vaccine, but the influenza vaccine has been implemented in Thailand for a long time with standard vaccine distribution. (3) The financial cost of COVID-19 vaccination might vary depending on health policy [22,23]. In Thailand, both the COVID-19 and influenza vaccines are free for the elderly and high risk populations [24]. However, our result showed that some patients did not know about their right to receive free influenza vaccines.

### 4.4. Public Health Implications

Influenza vaccination coverage should be aimed at WHO suggestions (75%). The Thai government, through the Ministry of Public Health, should set up policies and encourage greater coverage of vaccination in this group of patients as recommended by the WHO.

Pharmacists can play a role in identifying high-risk patients who have not yet received the vaccine and encouraging them to understand the importance of and desire for the influenza vaccine. Furthermore, community pharmacists can take on a role in improving accessibility to the vaccine and reducing vaccine hesitancy by educating and motivating patients and improving patient awareness and attitudes toward immunization. In the US and other high income countries, trained pharmacists have been authorized to administer vaccines, increasing immunization rates [25]. Still, pharmacists in Thailand do not play a role in vaccination [26]. Therefore, pharmacists’ collaborating with other healthcare providers is essential to improve opportunities and information for immunization across the public health system.

### 4.5. Limitations

Some limitations should be mentioned. First, the sample size was relatively small; however, this study provided the real-life practice of influenza vaccination among patients with chronic disease in northern Thailand. Second, this observational study may have been prone to bias due to interviewing methods, and elderly patients may be susceptible to recall bias. Nevertheless, two interviewers were trained to conduct patient interviews. Last, this study may have been limited to the situation before the 2019 novel COVID-19 outbreak. Thai citizens’ knowledge level and perceptions might have changed or improved [27] according to the findings from a meta-analysis that the COVID-19 pandemic improved worldwide intention to receiving the influenza vaccine [28].

## 5. Conclusions

The influenza vaccination coverage in northern Thailand among patients with DM or IHD was relatively low; less than one-half of patients received the influenza vaccination, but their knowledge level about influenza was high. Two factors associated with receiving influenza vaccination were being aware of the right to free vaccination and needing to receive the influenza vaccination. Thus, these factors should be improved to encourage patients with DM or IHD to receive the influenza vaccination annually to prevent cardiovascular events.

## Figures and Tables

**Table 1:** Patients’ characteristics by influenza vaccination status (n = 150).

Factor	Vaccinated (n = 68)	Not Vaccinated (n = 82)	*p*-Value
**Sex**			
Male	32 (47.1)	41 (50.0)	0.745
Female	36 (52.9)	41 (50.0)	
**Age**			
<65 years	26 (38.2)	43 (52.4)	0.100
≥65 years	42 (61.8)	39 (47.6)	
**Comorbidities**			
Diabetes mellitus	23 (33.8)	30 (36.6)	0.735
Ischemic heart disease	23 (33.8)	30 (36.6)	0.735
Diabetes mellitus and ischemic heart disease	22 (32.4)	22 (26.8)	0.477
**Highest education level**			
Lower than bachelor’s degree	58 (85.3)	61 (74.4)	0.110
Bachelor’s degree or higher	10 (14.7)	21 (25.6)	
**Influenza knowledge score (total of 11)**	9.91 ± 1.27	9.49 ± 1.40	0.056
10/11	49 (72.1)	47 (57.3)	0.087
<10	19 (27.9)	35 (42.7)	
**Perceptions and experiences on influenza vaccination**			
Knowing the risk of an existing disease	62 (91.2)	69 (84.2)	0.226
Knowing the advantages of being vaccinated	35 (51.5)	29 (35.4)	0.068
Knowing the disadvantages of being vaccinated	20 (29.4)	17 (20.7)	0.256
Fear of the complications of influenza vaccination	31 (45.6)	39 (47.6)	0.870
Knowing your right to receive free vaccination	52 (76.5)	43 (52.4)	0.004
Having a need to be vaccinated against the flu	57 (83.8)	48 (58.5)	0.001
Receiving influenza vaccination in the past	67 (98.5)	72 (87.8)	0.012

**Table 2:** Factors associated with influenza vaccination by univariable and multivariable logistic regression (n = 150).

Factor	Crude OR	95% CI	*p*-Value	Adjusted OR	95% CI	*p*-Value
**Personal factors**						
Female (reference: male)	1.13	0.59–2.14	0.720			
≥65 years (reference: <65)	1.78	0.93–3.42	0.083	1.91	0.93–3.92	0.079
Diabetes mellitus	0.89	0.45–1.74	0.725			
Ischemic heart disease	0.89	0.45–1.74	0.725			
Diabetes mellitus and ischemic heart disease	1.30	0.64–2.64	0.460			
Bachelor’s degree or higher (reference: lower than bachelor’s degree)	0.50	0.22–1.15	0.104			
**Knowledge score on influenza**						
High score: 10/11 (reference: low score: <10)	1.92	0.97–3.82	0.063	2.06	0.95–4.42	0.066
**Perceptions and experiences on influenza vaccination**						
Knowing the risk of an existing disease	1.95	0.70–5.43	0.203			
Knowing the advantages of being vaccinated	1.94	1.01–3.74	0.048	1.39	0.66–2.95	0.383
Knowing the disadvantages of being vaccinated	1.59	0.76–3.36	0.221			
Fear of the complications of influenza vaccination	0.92	0.48–1.76	0.809			
Knowing your right to receive free vaccination	2.95	1.45–5.99	0.003	2.32	1.06–5.10	0.035
Having a need to be vaccinated against the flu	3.67	1.68–8.01	0.001	3.50	1.51–8.12	0.003
Receiving influenza vaccination in the past	9.31	1.16–74.66	0.036	3.83	0.42–35.15	0.234

## Data Availability

The data presented in this study are available from the corresponding author on reasonable request.

## Supplementary Materials

The following supporting information can be downloaded at: https://www.mdpi.com/article/10.3390/vaccines11040794/s1, Supplement 1: Interviewing; Table S1: Patients’ knowledge score on influenza.
