# Supplementary material for: Influenza Vaccination among Patients with Diabetes or Ischemic Heart Disease in Thailand: Coverage, Knowledge and Associated Factors
Source: Vaccines (Basel). 2023 Apr 4;11(4):794. doi: 10.3390/vaccines11040794 (PMC10144710; doi:10.3390/vaccines11040794)
Supplement: Supplementary file 1 [file vaccines-11-00794-s001.zip › vaccines-2258043-supplementary.pdf]

## Supplement 1

## Interviewing

**The interview questions include four sections.**

**Part 1 – General information of participants**

1. Sex
   ▢ male ▢ female
2. Age …… years old
3. What is your highest education level?
   ▢ Lower than bachelor's degree
   ▢ Bachelor's degree or higher
4. Do you have any comorbidities?

| | | |
|---|---|---|
| Diabetes mellitus | ▢ yes | ▢ no |
| Ischemic heart disease | ▢ yes | ▢ no |
| Diabetes mellitus and ischemic heart disease | ▢ yes | ▢ no |

**Part 2 –Influenza vaccination**

1. Have you ever received an influenza vaccine in the past year?
   ▢ yes ▢ no

**Part 3 – Influenza knowledge**

Direction: Are these 11 items on the knowledge of influenza correct or not?

1. People who experience influenza will be able to spread the disease from the onset of symptoms.
   ▢ yes ▢ no
2. Influenza is mainly spread from person to person through droplets produced while coughing or sneezing.
   ▢ yes ▢ no
3. Influenza can be spread by sharing items with others such as bath towels, hand towels, food spoons, straws etc.
   ▢ yes ▢ no
4. Influenza cannot be contacted by touching hands, mucus, saliva and contaminated items such as doorknobs, handrails, telephones, tables, chairs etc.
   ▢ yes ▢ no
5. Transmission of influenza can be prevented mainly by wearing a face mask.
   ▢ yes ▢ no
6. Frequent hand washing with soap and water can help prevent influenza.
   ▢ yes ▢ no

7. Symptoms of influenza are the same as those of the common cold, e.g., high fever, muscle aches, cough, sore throat, runny nose, difficulty breathing, vomiting and diarrhea.
   ▢ yes ▢ no
8. If you become sick with influenza, you don’t need to take a break or stop studying.
   ▢ yes ▢ no
9. People should see a doctor if the fever has not reduced after 48 hours of presenting flu symptoms.
   ▢ yes ▢ no
10. People with medical conditions such as lung disease, asthma and diabetes need to see a doctor immediately if they have a cold.
   ▢ yes ▢ no
11. Maintaining good health by exercising regularly, eating healthful food, and getting sufficient rest can help prevent influenza.
   ▢ yes ▢ no

**Part 4 - Factors associated with influenza vaccination.**

Direction: Please answer each question based on your perceptions and experiences on influenza vaccination.

1. Are you aware that the heart disease and/or diabetes you have can cause you to be re-hospitalized or have an increased risk of death from the onset of the disease?
   ▢ yes ▢ no
2. Are you aware of the advantages of seasonal influenza vaccination, such as preventing severe complications when influenza flare-ups occur?
   ▢ yes ▢ no
3. Are you aware of the disadvantages/side effects of receiving the vaccine? For example, it may cause allergies, fever, chills, muscle pain at the vaccination site, etc..
   ▢ yes ▢ no
4. Are you afraid of vaccine complications, such as side effects from getting the influenza vaccine?
   ▢ yes ▢ no
5. Did you know that you have the right to receive free vaccination?
   ▢ yes ▢ no
6. Do you need to be vaccinated against the flu?
   ▢ yes ▢ no
7. Have you received a seasonal influenza vaccine in the past few years?
   ▢ yes ▢ no

**Table S1.** Patients' knowledge score on influenza (n = 150)

| Knowledge score on influenza | Patients with correct answer | | |
| --- | --- | --- | --- |
| | Vaccinated (n = 68) n (%) | Not vaccinated (n = 82) n (%) | *p*-value |
| 1. People who experienced influenza will be able to spread the disease from the onset of symptoms. | 61 (89.7) | 69 (84.1) | 0.346 |
| 2. Influenza is mainly spread from person to person through droplets produced while coughing or sneezing. | 65 (95.6) | 76 (92.7) | 0.512 |
| 3. Influenza can be spread by sharing items with others, such as bath towels, hand towels, food spoons, straws etc. | 62 (91.2) | 73 (89.0) | 0.787 |
| 4. Influenza cannot be contacted by touching hands, mucus, saliva and contaminated items such as doorknobs, handrails, telephones, tables, chairs etc. | 55 (80.9) | 61 (74.4) | 0.434 |
| 5. Transmission of influenza can be prevented mainly by wearing a face mask. | 66 (97.1) | 81 (98.8) | 0.590 |
| 6. Frequent hand washing with soap and water can help prevent influenza. | 65 (95.6) | 78 (95.1) | 1.000 |
| 7. Symptoms of influenza are the same as those of the common cold, e.g., high fever, muscle aches, cough, sore throat, runny nose, difficulty breathing, vomiting and diarrhea. | 48 (70.6) | 50 (61.0) | 0.233 |
| 8. If you become sick with influenza, you don't need to take a break or stop studying. | 52 (76.5) | 57 (69.5) | 0.364 |
| 9. People should see a doctor if the fever has not reduced after 48 hours of presenting flu symptoms. | 67 (98.5) | 76 (92.7) | 0.128 |
| 10. People with medical conditions such as lung disease, asthma and diabetes need to see a doctor immediately if they have a cold. | 66 (97.1) | 77 (93.9) | 0.457 |
| 11. Maintaining good health by exercising regularly, eating healthful food, and getting sufficient rest can help prevent influenza. | 67 (98.5) | 80 (97.6) | 1.000 |
| Influenza knowledge score (total of 11) | 9.91 ± 1.23 | 9.49 ± 1.40 | 0.056 |
| Score 10/11 | 49 (72.1) | 47 (57.3) | 0.087 |
| Score <10 | 19 (27.9) | 35 (42.7) | |
